# Brain resilience to targeted attack of resting BOLD networks as a measure of cognitive reserve

**DOI:** 10.1162/IMAG.a.1065

**Published:** 2026-01-13

**Authors:** Georgette Argiris, Yaakov Stern, Christian Habeck

**Affiliations:** Cognitive Neuroscience Division, Department of Neurology, Columbia University Irving Medical Center, New York, NY, United States; Taub Institute, Columbia University, New York, NY, United States

**Keywords:** cognitive aging, RANN, CR, targeted network attack, resting-state functional connectivity, longitudinal

## Abstract

Recent advancements in connectome analyses have enabled more precise measurements of brain network integrity. Identifying neural measures that can operate as mechanisms of cognitive reserve is integral for the study of individual variability in age-related cognitive changes. In the present study, we tested the hypothesis that network resilience, or the network’s ability to maintain functionality when facing internal or external perturbations that cause damage or error, can function as a cognitive reserve (CR) candidate, modifying the relationship between cognitive and brain changes in a lifespan cohort of cognitively healthy adults. One hundred cognitively healthy older adults from the Reference Ability Neural Network (RANN) longitudinal lifespan cohort (50–80 years) underwent resting-state fMRI and neuropsychological testing at baseline and 5-year follow-up. Using undirected weighted adjacency matrices created from the Schaefer et al. (2018) 400-parcellation atlas and 19 additional subcortical regions (419 nodes in total), whole-brain network resilience was assessed through a targeted attack approach, where nodes were sequentially removed by nodal strength and resilience defined as the iteration of the steepest slope in the largest connected component (LCC) decay. We observed that network resilience moderated the effect of cortical thickness (CT) changes on longitudinal changes in Fluid Reasoning performance, even after adjusting for baseline differences, demographic factors, and the initial LCC of the unlesioned matrix, indicating that individuals with greater resilience were less sensitive to the effect of cortical thickness changes on changes in cognition. These findings support the use of targeted attack as a measure of cognitive reserve, suggesting that higher network resilience may allow individuals with reduced brain integrity to better cope with structural loss and maintain cognitive function.

## 1. Introduction

The concept of cognitive reserve (CR) addresses individual variability in age-related cognitive outcomes, whereby some individuals withstand greater levels of brain pathology than others before manifesting cognitive decline. A recent research consortium created a consensus definition of CR as a property of the brain that allows for cognitive performance that is better than expected given the degree of life-course-related brain changes and brain injury or disease (Stern et al., 2023). This property of the brain can refer to multiple potential mechanisms, from the molecular- to network-level and should explain cognitive performance beyond the influence of brain status, the latter two ideally measured longitudinally. Quantifying at least one aspect of brain integrity is a core tenet of the CR framework to identify a genuine mechanism of CR, ensuring it is not confounded by differences in the brain’s neurobiological status that might support better cognition. The neural implementation of CR can be investigated from multiple perspectives, with prior research evidencing a task-activation-invariant CR network (Stern et al., 2018) and resting-BOLD functional connectivity pattern linked to higher IQ (Stern et al., 2021). In the current work, we explore whether a functional connectome’s resilience to network attack operates as a mechanism of CR, moderating the impact of brain change on cognitive change.

The human connectome constitutes the structural and functional network of connections within the human brain and has been described as a complex network (Sporns et al., 2005). Similar to other complex networks found in nature (e.g., social networks, the world wide web, or biological systems), the human connectome exhibits various properties that characterize complex systems, such as highly structured connectivity patterns, multiscale dynamics, small-world properties that reflect optimized information flow, and resilience to external perturbations (Albert et al., 2000; Sporns, 2011; Strogatz, 2001; Watts, 2004). Graph theory has proven to be one important framework for characterizing properties of complex networks. When applied to the topology of the brain, graphs are constructed of nodes, which represent distinct brain regions, and edges, which are their structural or functional couplings relating to information flow (Bullmore & Bassett, 2011). Then, a series of network properties, or graph metrics, can be calculated to provide insight into the configuration of the network and assess strengths and weaknesses of that configuration.

With advancements in brain connectome analyses, it is possible to obtain more fine-grained measurements of brain network integrity. One measure of integrity is resilience, or the capacity of the network to retain functionality when confronted with endogenous or exogenous perturbations that result in damage or error (Gao et al., 2016). Maintenance of specific structural or functional connectivity patterns despite the deleterious effects of aging and disease is a core feature of brain network resilience; in the highly resilient brain, a network should resist disconnection or disintegration in its topology even when key brain regions are attacked (Stanford et al., 2022). To obtain network resilience estimates, virtual “lesions” are typically induced via in silico simulations, wherein nodes or edges are sequentially removed based on some pre-determined metric of significance to the network (Achard et al., 2006; Aerts et al., 2016; Joyce et al., 2013).

Computational modeling of lesion effects in the human brain has drawn a link between localized structural damage of networks and global network disruptions that could impact behavioral and cognitive outcomes (Alstott et al., 2009). Preservation of specific functional network metrics, such as global system segregation and modularity (Ewers et al., 2021; Varangis et al., 2021), local efficiency (Adams et al., 2023), and robustness of core network topology (Stanford et al., 2022) has been linked to cognitive resilience in both healthy aging and Alzheimer’s disease (AD). Prior work has also displayed a link between network integration measured across graph filtration thresholds and fluid reasoning performance in a longitudinal aging cohort (Argiris et al., 2023). Investigating dynamic changes of network topology through iterative processes, emphasizing metrics related to nodal importance, could provide valuable insights into the mechanisms underlying cognitive changes. Moreover, the brain’s capacity to retain network functionality and resist perturbations may serve as a potential mechanism of CR, wherein individuals with higher network resilience may better resist the pathological impacts of brain “hardware” malfunctions and sustain higher level of cognitive functioning (Stern, 2009; Stern et al., 2023).

Here, we investigated whether a measure of network resilience, based on in silico lesioning of the largest connected component (LCC) of the network, can possibly serve as a mechanism of CR in healthy older adults. Importantly, the largest connected component (LCC) does not simply reflect stronger overall connectivity but captures a distinct aspect of global network integrity: the capacity of a brain network to maintain large-scale interconnectedness under perturbation. As nodes are removed in a targeted attack, the breakdown of the LCC marks the fragmentation of the network into disconnected components, potentially impairing communication between regions. Thus, the preservation of the LCC across attack iterations may reflect a form of functional redundancy or topological robustness—both of which align with the concept of CR as the ability to sustain healthy cognition in the face of neural degradation. Unlike mean connectivity, which quantifies average link strength, LCC resilience reflects how well a network preserves global coherence under structural loss, making it particularly relevant to CR models focused on adaptive brain network function (Bullmore & Sporns, 2012; Fornito et al., 2015). Recent prior work has demonstrated the LCC as a measure of structural network integrity (Alstott et al., 2009; Joyce et al., 2013; Watts & Strogatz, 1998). Additionally, Menardi et al. (2021) analyzed brain network resilience of LCC to targeted attack in twins and found moderate heritability several topological network measures, including the critical point in the drop of the LCC. In the current work, we wanted to establish to what extent the critical point of drop over lesioning iterations can offer predictive utility in explaining cognitive change beyond the information encoded in the static connectome.

We utilized longitudinal resting-state and neuropsychological data from the Reference Ability Neural Network (RANN) and Cognitive Reserve (CR) cohorts to test for moderation between changes in structural brain integrity and network resilience as it relates to out-of-scanner task performance. We used cortical thickness (CT) as our longitudinal measure of brain integrity, described in detail in section 2.2.5. The key research question was whether network resilience, as a potential mechanism of cognitive reserve (CR), moderates the relationship between changes in CT and changes in cognitive performance. CT has been shown to decline throughout the lifespan (Fjell et al., 2015) and is a predictor of cognitive performance (Dominguez et al., 2021). Additionally, cross-sectional research from our lab has demonstrated negative associations between age and CT, while also uncovering a complex relationship between CT and other demographic factors (Habeck et al., 2020). Finally, in order to balance network properties such as efficiency and robustness, brain networks exhibit distinctive configurations that enable efficient local processing and global integration of their components (Deco et al., 2015; Rubinov & Sporns, 2010) to ensure high levels of resilience (Achard et al., 2006; Joyce et al., 2013). As small-world organization is thought to reflect this balance (Albert et al., 2000; Watts, 2004), we also tested its relationship to the LCC and behavior. Ultimately, we hypothesized that network resilience—operationalized as the ability to retain larger LCCs across successive iterations of in silico lesioning—would moderate the relationship between changes in cortical thickness and changes in cognitive performance, such that individuals with more resilient networks would be less susceptible to the cognitive consequences of age-related structural brain changes.

## 2 Methods

### 2.1 Participants

Analyses included one hundred native English-speaking, right-handed (Oldfield Edinburgh Handedness Inventory; Oldfield, 1971) older adults (age = 64.22 ± 7.33; range = 50 - 80 years) from the Reference Ability Neural Network (RANN) or the Cognitive Reserve (CR) cohort, both community-based cohort from the greater New York area. Both cohorts employed identical inclusion/exclusion criteria, structural and resting state functional imaging protocols, as well as a considerable overlap in cognitive assessments and questionnaires, with some participants belonging to both cohorts (=36 participants). The main contrast between the two studies lies in the functional task-based imaging protocols, which will not be elaborated on here since no task-based imaging is utilized. No significant group differences were observed between participants belonging to either cohort or both cohorts with respect to Age (p = .635), Sex (p = .333), Education (p = .899), or NART (p = .656). A brief overview of task-based imaging protocols for CR in-scanner tasks can be found in Habeck et al. (2022). Participants were tested at two time points— baseline and 5-year follow-up. Participants were screened for psychiatric and medical conditions, uncorrectable hearing and vision loss, and any other contraindications that could have impeded MRI acquisition prior to study participation. Participants were tested at both time points for dementia and mild cognitive impairment using the Dementia Rating Scale (DRS; Mattis, 1988). All participants had less than 30% of their functional data removed and interpolated (i.e., scrubbed; see section 2.2.2.2. Data Processing) due to motion artifact (Parkes et al., 2018; Power et al., 2012). See Table 1 for a list of participant demographics.

**Table 1.** Participant demographics divided by decade of life.

		Sex		Age (years)		NART		Education (years)	
Age bracket	N	Male	Female	M	SD	M	SD	M	SD
50–59 years	24	10	14	54.08	3.05	119.49	7.54	15.79	2.15
60–69 years	52	29	23	64.77	2.78	119.34	7.69	16.02	2.36
70–80 years	24	14	10	73.46	2.70	119.23	8.35	16	2.36

Mean (M) and standard deviation (SD) are presented for age, the National Adult Reading Test (NART), and Education.

### 2.2 Procedure

Participants were scanned during a period of rest prior to the task-based imaging protocol during which cognitive tasks were performed in-scanner. Only resting state data will be considered here. Participants additionally underwent neuropsychological assessment in a prior session. The relationship between out-of-scanner neuropsychological performance and resting-state connectivity will be analyzed.

#### 2.2.1 Neuropsychological test assessment

All participants underwent an out-of-scanner comprehensive neuropsychological battery to assess an array of cognitive functions. The tasks were administered in the following fixed sequence: Wechsler Adult Intelligence Scale (WAIS-III; Wechsler, 1997), Letter-Number Sequencing, American National Adult Reading Test (AMNART; Wechsler, 1997), Selective Reminding Task (SRT) immediate recall (Buschke & Fuld, 1974), WAIS-III Matrix Reasoning (Wechsler, 1997), SRT delayed recall and delayed recognition (Buschke & Fuld, 1974), WAIS-III Digit Symbol (Wechsler, 1997), Trail-Making Test versions A and B (TMT-A/B; Reitan, 1978), Controlled Word Association (C-F-L) and Category Fluency (animals; Benton et al., 1983), Stroop Color Word Test (Golden, 1975), Wechsler Test of Adult Reading (WTAR; Holdnack, 2001), WAIS-III Vocabulary (Wechsler, 1997), and WAIS-III Block Design (Wechsler, 1997).

Previous analyses demonstrated that the administered tasks represent distinct latent variables for four cognitive domains (Razlighi et al., 2017; Salthouse & Ferrer-Caja, 2003). The four cognitive domains represented were: Episodic Memory (three measures from the SRT); Fluid Reasoning (WAIS Matrix Reasoning, WAIS Block Design, and TMT-B); Perceptual Speed (WAIS Digit Symbol, 2 measures from the Stroop test, TMT-A); and Vocabulary (WAIS Vocabulary, AMNART, WTAR). To standardize comparisons between tasks, behavioral scores at each time point were z-transformed using the mean and standard deviation calculated across all participants for each task separately at baseline. Since speed tasks were measured as reaction time, z-scores were inverted so that higher scores always indicate better performance. The three tasks belonging to each domain were then averaged to generate a composite domain score.

#### 2.2.2 fMRI data acquisition

##### 2.2.2.1 Scan parameters

Image acquisition was performed using a 3T Philips Achieva Magnet; thus, scanner parameters were consistent across participants and sessions. The resting state protocol consisted of fMRI scans collected for a period of either 5 minutes (baseline: *n* = 46; follow-up: *n* = 3) or 9.5 minutes (baseline: *n* = 54; follow-up: *n* = 97) minutes, with eyes closed. A T1-weighted image of the whole brain was performed for each subject with a Magnetization Prepared Rapid Gradient Echo (MPRAGE) sequence with the following parameters: TE/TR of 3/6.5 ms, flip angle of 8°, in-plane resolution of 256 × 256 voxels, field of view (FOV) of 25.4 × 25.4 cm, and 165–180 slices in the axial direction with a slice-thickness/gap of 1/0 mm. All scans used a 240 mm field of view. For the fMRI blood oxygen level-dependent (BOLD) resting-state scans, the following parameters were used: TE/TR of 20/2000 ms, flip angle of 72°, in-plane resolution of 112 × 112 voxels, and a slice thickness/gap of 3/0 mm and 37 slices. A neuroradiologist analyzed each participant’s scan for any anatomical anomalies. Participants with clinically significant anomalies were excluded from the study. Any noteworthy observations were communicated to the participant’s primary healthcare provider in accordance with ethical reporting procedures.

##### 2.2.2.2 Data processing

Images were preprocessed using an in-house developed native space method (Razlighi et al., 2014). In brief, the preprocessing pipeline was as follows: slice timing and motion correction (MCFLIRT) were applied using the FSL package (Jenkinson et al., 2012). Registration to the middle volume was performed with 6 degrees of freedom, 256 bins mutual information as the cost function (Jenkinson & Smith, 2001), and sinc interpolation. Frame-wise displacement (FD) and root-mean-square difference (RMSD) were computed from motion parameters and BOLD percentage signal, with a conservative RMSD threshold of 0.3%. Detected contaminated volumes, meeting criteria of FD > 0.5 mm or RMSD > 0.3%, were replaced by linearly interpolated adjacent volumes prior to temporal filtering (Carp, 2013). We utilized a 0.5 mm FD threshold for motion scrubbing, consistent with Power et al. (2012) and widely adopted in aging cohorts, although stricter thresholds (e.g., 0.2–0.3 mm) have been considered in later work (e.g., Ciric et al., 2017; Power et al., 2014). Because FD magnitudes scale with TR—longer TRs yield larger per-frame displacements for the same physical motion—this cutoff is effectively more conservative in our 3T, TR = 2 second acquisition than in short-TR multiband datasets, such as the Human Connectome Project. The 0.5 mm cutoff remains a common pragmatic choice, especially in older adult cohorts where stricter thresholds can compromise data retention. Moreover, our focus on relative topological properties (e.g., nodal rankings and LCC critical points), rather than absolute connectivity values, reduces direct susceptibility to distance-dependent shifts from residual motion. Each interpolated volume was flagged, and the resulting scrubbing percentage was used to exclude participants with excessive artifacts (>30% scrubbed volumes). Subsequently, motion-corrected signals underwent bandpass filtering (0.01 to 0.09 Hz) using Flsmaths–bptf. Finally, data were residualized by regressing out FD, RMSD, white matter signals, and lateral ventricular signals (Birn et al., 2006). Each T1-weighted structural image was aligned to the 2 mm MNI template using advanced normalization tools (ANTs) nonlinear registration, and the mean functional image was registered to the T1 image using FLIRT with 6 degrees of freedom and mutual information.

#### 2.2.3 Functional connectivity

**Fig. 1.**
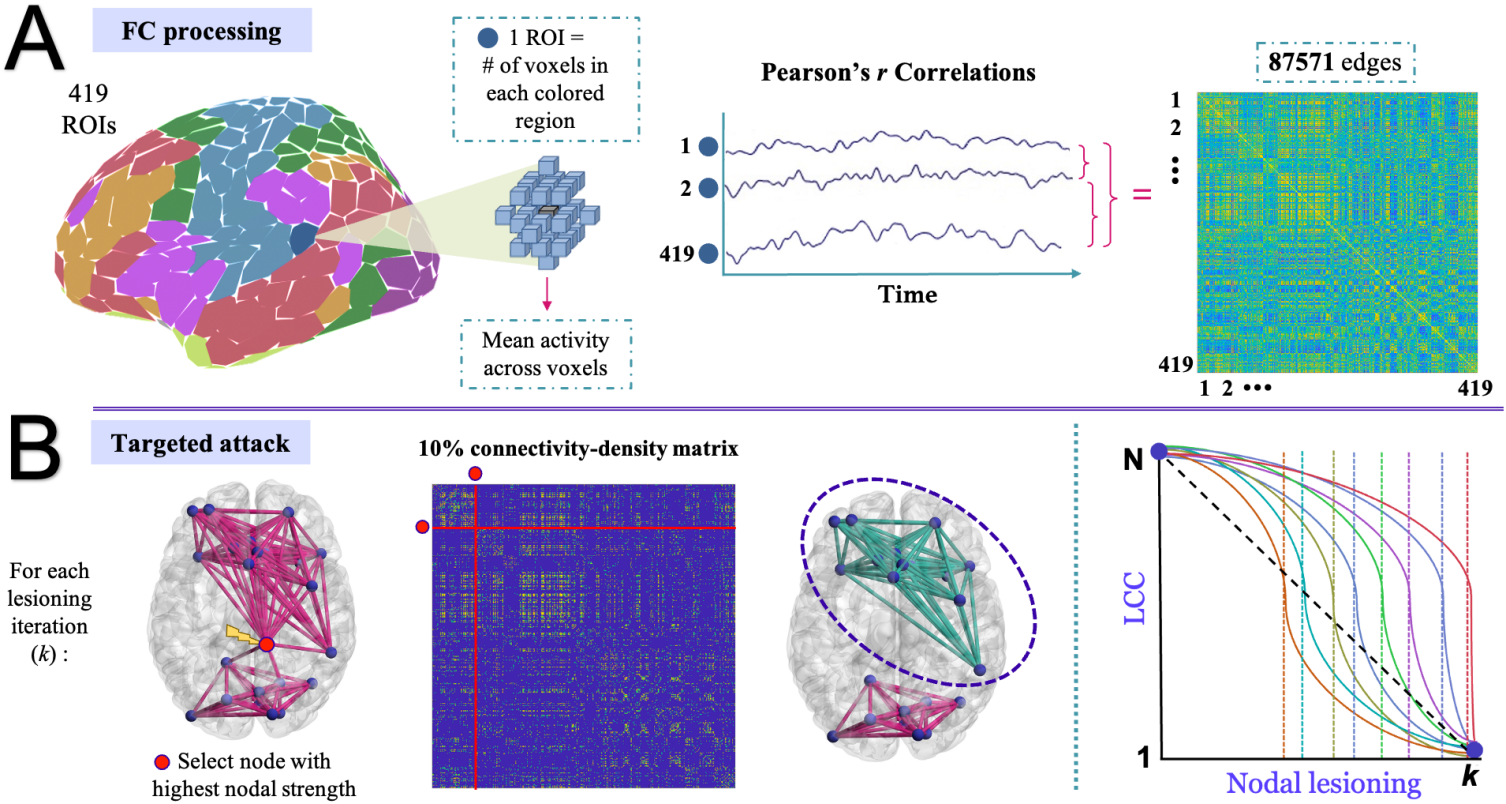
Schematic of functional connectivity (FC) matrix generation and application of targeted attack. (A) Time-series extraction for each region of Schaefer’s 400 parcellation scheme in addition to the 19 subcortical regions obtained from FreeSurfer segmentation. Time-series data were averaged across all voxels within each region, and a cross-correlation connectivity matrix using Pearson’s *r* was created from all nodal pairings. Brain parcellation image was created with the ggsegSchaefer library in R (Mowinckel & Vidal-Piñeiro D, 2022). (B) Each participant’s connectivity matrix was thresholded at a 10% connection density to retain the strongest (positive) connections. In the toy brain example, the complete network (left) represents the largest connected component (LCC). The node with the highest nodal strength is identified and removed (red), and the resulting cluster (green) represents the new LCC. The plot on the far right shows potential curves across nodal lesioning iterations (k = N, where N is the maximum number of nodes, 419). Each colored curve represents an individual, with colored dashed lines indicating possible critical drop-off points or maximum deflections, where the number of lesioning iterations sustained distinguishes between a low resilient individual (i.e., orange curve/dashed line) versus a high resilient individual (red curve/dashed line). The dashed diagonal black line merely indicates hypothetical LCC drop under random attack, which was not focused here.

T1 image segmentation was performed using FreeSurfer (Dale et al., 1999). We utilized the Schaefer atlas with 400 parcels, which divides the cortex into 400 distinct regions based on functional and anatomical features. Several parcellation schemes exist for the Schaefer atlas, ranging from 100 to 1000 parcels and divided into either 7 or 17 functional networks. Given that there is an estimated 300–400 defined human neocortical areas (Van Essen et al., 2012), we opted for the 400-ROI parcellation scheme for greater precision, as it has also been suggested that lower-resolution parcellations may not be able to fully differentiate cortical areas (Gordon et al., 2016; see Schaefer et al., 2018). Additionally, 19 subcortical grey matter structures were added, as described by Glasser et al. (2016) and considered in previous literature (Li et al., 2019). These 19 subcortical regions were defined by FreeSurfer (Fischl et al., 2002) and correspond to the cerebellar gray matter, thalamus, caudate, putamen, pallidum, hippocampus, accumbens, amygdala, ventral diencephalon, and brain stem. Thus, a total of 419 regions-of-interest (ROIs) were considered. The 419-region Schaefer parcellation (including subcortical nodes), defined in MNI space, was then applied directly to the fMRI data in MNI space. A subject-specific gray matter mask (derived from FreeSurfer’s segmentation, dilated to account for registration imprecision, and transformed to MNI space using the same warp fields) was used to constrain the extraction to gray matter voxels. This dilated mask provides a slight expansion around the gray matter boundary to account for small inaccuracies during the segmentation or registration process. An intermodal, intra-subject, rigid-body registration of the fMRI reference image and T1 scan was performed with FLIRT with 6 degrees of freedom and normalized mutual information and was used as part of the transformation chain to bring the fMRI data into MNI space. All voxels within each nodal mask were averaged to obtain a single fMRI time-series per node. Pearson correlations were then performed for all pairwise nodal combinations, which resulted in 419 × (418/2) = 87,571 fMRI connectivity pairs (see Fig. 1A for FC schematic). Pearson correlation was computed across the time series data, including only volumes that were not interpolated. Thus, although interpolated volumes were retained as placeholders for preprocessing continuity, they were excluded from the final construction of the correlation matrices. Cross-correlation matrices of time series were derived without regressing out the global signal, as this procedure is known to alter correlation patterns and magnitudes (Alstott et al., 2009). Although global signal regression (GSR) can enhance certain associations—such as increasing brain–behavior correlations by 40–60% in some contexts (Li et al., 2019)—its use remains controversial. The global signal has been shown to contain neuronal information (Schölvinck et al., 2010) and to reflect fluctuations in arousal and vigilance (Wong et al., 2016), prompting concerns that GSR may remove meaningful neural signals (Glasser et al., 2018). Moreover, while GSR can reduce correlations between quality control metrics and functional connectivity, this effect is distance-dependent and may introduce spatial biases (Parkes et al., 2018). Finally, matrices were not Fisher Z-transformed because the graph theory metrics were based on distance calculations, which constrain values to a range between 0 and 1.

#### 2.2.4 Network measures

Prior to targeted attack, participant matrices underwent density-based thresholding to retain only the strongest connections. Previous work has shown measures of network topology to be sensitive to different types of thresholding (van Wijk et al., 2010), in addition to age-related effects emerging only in certain network metrics and range of connectivity thresholds (Geerligs et al., 2015; Iordan et al., 2018; Varangis et al., 2021). In the current analysis, we adopted a primary connectivity-density threshold of 10% for several reasons (1) this threshold falls within the range used in previous studies; (2) it was employed in the targeted attack study by Menardi et al. (2021); and (3) a 10% sparsity threshold has been associated with higher test-retest reproducibility for global network metrics (Wang et al., 2011). However, as a sensitivity analysis, we also repeated the analysis using two adjacent thresholds, 5% and 15%, around the 10% threshold. While these additional thresholds were intended to confirm the robustness of our findings, we did not systematically explore a broader range, as thresholding inherently involves making assumptions about which connections are meaningful. Beyond certain sparsity levels, invariance in network properties cannot be assumed, and differences are expected—reflecting the conceptual basis of thresholding itself. A 10% density threshold was applied to each participant’s individual connectivity matrix (nodes surviving threshold—baseline: M* = *415.21, SD = 5.72; follow-up: M *=* 414.45, SD = 8.97). As the 10% threshold was defined as the primary analytical focus and additional thresholds were examined solely to assess the stability of this finding, we did not apply a correction for multiple comparisons.

For each individual’s connectivity matrix, three graph theory metrics were extracted using Brain Connectivity Toolbox (Rubinov et al., 2009) functions implemented in MATLAB:

1. *clustering coefficient*: a measure of network segregation, defined as the ratio between the number of connections that exist between direct neighbors of a node and the maximum number of possible connections, averaged across all network nodes.
2. *characteristic path length*: a measure of integration, defined as the average shortest path length (i.e., minimum number of connections to link two nodes) across all network pairs
3. *largest connected component (LCC)*: defined as the component of the graph that contains the largest number of nodes that are connected by edges.

For each participant’s original connectivity matrix, we also generated random matrices that preserved the number of nodes and degree distribution, following the algorithm introduced by Maslov and Sneppen (2002). In this procedure, pairs of edges are randomly selected and their connections are swapped (i.e., rewired), provided that the rewiring does not create multiple edges between the same pair of nodes or self-loops. This process ensures that, while the specific pattern of connectivity is randomized, each node retains its original degree (i.e., the same number of connections as in the empirical network), thereby preserving the degree distribution of the network while disrupting the higher-order structure. To ensure sufficient randomization, we repeated the edge-swapping procedure so that each edge was rewired over 100 iterations. The resulting randomized matrices served as null networks, allowing us to normalize graph theory metrics by comparing the original network values to those expected by chance, given the same degree distribution. The clustering coefficient and characteristic path length of the original matrices, normalized by the values derived from random matrices, were used to calculate the small-world network property of each participant’s graph *prior* to lesioning. It was computed as follows:

1. Let CPL[p] the characteristic path length, and CC[p] the clustering coefficient for each participant *p*. Let $CC_{\text{rand}}\;$ represent the clustering coefficient and $CPL_{\text{rand}}$ the characteristic path length of the randomized matrix.
2. Define SW[p] as the small-world network expressed as:

$$SW\left[p\right]\;=\frac{CC(\left[p\right])\;/CC_{\text{rand}}\left[p\right]}{CPL(\left[p\right])\;/CPL_{\text{rand}}\left[p\right]}$$

Network resilience was computed via the sequential removal of nodes from the weighted adjacency matrix, based on prior lesioning studies (e.g., Albert & Barabási, 2002; Albert et al., 2000; Alstott et al., 2009; Menardi et al., 2021).

##### 2.2.4.1 Targeted network attack

For the targeted attack sequence, nodes were sorted in descending order based on their nodal strength. We chose nodal strength as the organizing principle to preserve more information by summing the weights across all edges, rather than simply binarizing to obtain the cardinal quantity of connections. However, Alstott et al. (2009) demonstrated that targeted attack by either degree or strength produce almost identical results.

At each iteration of attack, nodal strength was calculated to account for the effect of prior lesioning and the node with the highest nodal strength, along with all of its connections, was removed from the graph; graph theory metrics were then extracted. The sequence of steps can be expressed as follows:

1. Let A be the N × N connectivity matrix where $A_{ij\;}$ represents the Pearson’s *r* connectivity value between node *i* and node j.
2. Calculate each graph theory metric on the initial connectivity matrix where $A^{\left(0\right)}$ = *A*.
3. For each iteration *k* from 1 to N:
  - Calculate the nodal strengths for each node *i* in the matrix $A^{\left(k-1\right)}$ :
    $$s_{i}^{\left(k-1\right)}=\sum_{j\in I}A_{ij}^{\left(k-1\right)}$$
    where *I*_*k*−1_ is the set of indices of the remaining nodes in $A^{\left(k-1\right)}$ .
  - Find the node $n_{k}$ with the highest nodal strength:
    $$n_{k}=\underset{j\in I_{k-1}}{\arg\;\max}s_{i}^{\left(k-1\right)}$$
  - Update the set of remaining nodes with the removal of $n_{k}$:
    $I_{k}\;=I_{k-1}\text{​}\backslash \text{​}\{n_{k\;}\}\;$
  - Update the connectivity matrix by removing the rows and columns corresponding to $n_{k}$:
    $$A^{\left(k\right)}=\;A^{\left(k-1\right)}\left(I_{k},\;I_{k}\right)$$
  - Calculate each graph theory metric on the updated matrix.

Our primary measure of network resilience was based on the rate of change in the LCC of the graph. In a fully connected graph, the LCC initially includes all the nodes; however, as nodes are iteratively removed, the graph becomes increasingly fragmented until the LCC is reduced to a single node (the last remaining node; see Fig. 1B). We inferred that more resilient individuals would sustain larger LCCs over longer iterations of lesioning before decay in LCC becomes evident (i.e., critical point), with resilience operationalized as the iteration of steepest slope drop in LCC. Our primary planned analysis of the LCC under targeted attack did not include normalization against random graphs, as the initial goal was to quantify the direct impact of targeted attack on the empirical network; absolute resilience of the network is measured without introducing unnecessary complexity.

The critical point was calculated as follows:

1. Let lcc [*p*, *i*] be the value of the LCC for participant *p* at iteration *i*.
2. Define $LCC_{\text{slope}}$ [*p*, *i*] as the slope between points *i* and *i* + 1 for participant *p*.
3. Calculate the slope for each participant *p* and each point *i*:
  $$LCC_{\text{slope}}\text{​}\left[p,i\right]\;=lcc\left[p,i+1\right]\;-\;lcc\left[p,i\right]$$
4. Define $LCC_{\text{drop }}$ as the minimum slope for each participant *p* across the *n* node set:
  $$LCC_{drop}[p]=\min(LCC_{siope}[p,i])\;for\;i\in \{1,2,\ldots ,n-1\}i$$

In line with prior work examining targeted attack in brain networks (e.g., Achard et al., 2006; Alstott et al., 2009), we report unnormalized LCC curves as a main analysis. Our objective was not to quantify absolute LCC values or compare them against random topology, but rather to assess subject-specific patterns of network vulnerability. Normalization against randomized graphs may obscure biologically meaningful individual differences by removing topological features that are functionally relevant. However, in response to reviewer comments, we additionally performed targeted attack on the randomized matrices (see section 2.2.4), constructed LCC curves from these randomized matrices, and computed a normalized LCC metric. Normalization was performed by dividing the empirical LCC curves by the normalized LCC curves. These analyses were conducted post hoc to ensure that our findings were not driven by trivial network properties and to allow direct comparison of empirical results to appropriate random baselines (for results, see Supplementary Table ST1).

##### 2.2.4.2 Random network attack

To complement the targeted attack analysis and to demonstrate the specificity of the observed effects, we also performed a random network attack procedure. This additional analysis served as a critical control, helping to determine whether the patterns identified in the targeted attack reflect meaningful differences in network resilience—or whether they could be explained by general, non-specific vulnerabilities inherent in all networks. For each participant, a random vector was generated to determine the sequence of node removal. Nodes were iteratively removed from the network based on this randomized order, and the size of the LCC was recalculated at each step. This analysis was conducted for visualization and interpretive purposes only; random attack metrics were not included in statistical modeling, as our primary focus was on the network’s resilience to targeted, biologically meaningful disruptions.

#### 2.2.5 Cortical thickness (CT)

CT measurements were obtained from each participant’s T1-weighted MPRAGE image using the FreeSurfer software package (v5.1.0) (http://surfer.nmr.mgh.harvard.edu/). While the estimation process was automated, segmentation of gray and white matter, as well as spatial registration, was manually reviewed following the guidelines provided by Fjell et al. (2009). Since the dataset included longitudinal data across two time points, images were processed through the longitudinal pipeline (Reuter et al., 2012). This approach generates an unbiased within-subject template space and image using inverse consistent registration (Reuter et al., 2010). All subsequent processing steps leverage shared information from this within-subject template, a method demonstrated to enhance both statistical power and reliability (Reuter et al., 2012). CT was estimated by first reconstructing the gray/white matter boundary and cortical surface (Dale et al., 1999). The thickness at each point along the cortex was then calculated as the shortest distance between the gray/white matter boundary and the outer cortical surface. Finally, mean CT was computed across the entire cortex, resulting in a single representative value.

#### 2.2.6 Analytic approach

Linear regression analyses with change score models were utilized. Age, NART IQ (NART), education (Edu), sex, and CT were included as covariates in all regression models. Scrubbing percentage was included as a nuisance variable in all models. We did not include mean FD because scrubbing percentage is derived directly from FD; including both would introduce redundancy and potential collinearity without adding unique information. Indeed, the correlation between scrubbing percentage and mean FD in our sample was r = 0.90. Our primary variable of interest was the interaction between cortical thickness (CT) and network resilience, tested as a potential marker of cognitive reserve (CR). We tested network resilience as a measure of CR and variable of interest by utilizing the $LCC_{\text{drop}}$ at follow-up, or time 2 ($LCC_{{\text{drop}}^{\text{T}2}}$ ) as it represented the most recently acquired indicator of brain status and a potential marker of CR. Due to changes in the acquisition protocol over the course of the study, nearly half of the participants at baseline were scanned for only 5 minutes, while the longer 9.5-minute protocol was implemented during later follow-up sessions. For this additional reason, we chose to use follow-up data, as a greater proportion of participants had longer scan durations, providing a more reliable basis for functional network characterization. Although the reliability of resting-state network metrics is known to be lower at shorter scan lengths (Birn et al., 2013), previous work indicates that extending scan duration from 5 to 13 minutes improves reliability, and that in samples of 100 or more participants, reliable global and regional graph metrics can be obtained with approximately 7 minutes of data (Termenon et al., 2016). As an additional check, we conducted a sub-analysis using truncated 5-minute time series, with results reported as Supplementary Table ST1.

For regressions of longitudinal change, change values were calculated as follow-up (FU) minus baseline (BL). For change in CT (ΔCT), the change values were residualized with respect to baseline measurements. For behavioral performance, change values were not residualized with respect to baseline, as we were interested in explicitly modeling baseline effects.

We examined the association between network resilience (i.e., $LCC_{{\text{drop}}^{\text{T}2}}$ ) and four cognitive domains selected a priori, each representing a theoretically distinct construct. Based on prior structural equation modeling work (e.g., Salthouse et al., 2009; Stern et al., 2014), these domains are considered orthogonal and were treated as independent outcomes. Accordingly, no correction for multiple comparisons was applied, as each model tested a separate hypothesis rather than a family of related tests.

## 3 Results

**Fig. 2.**
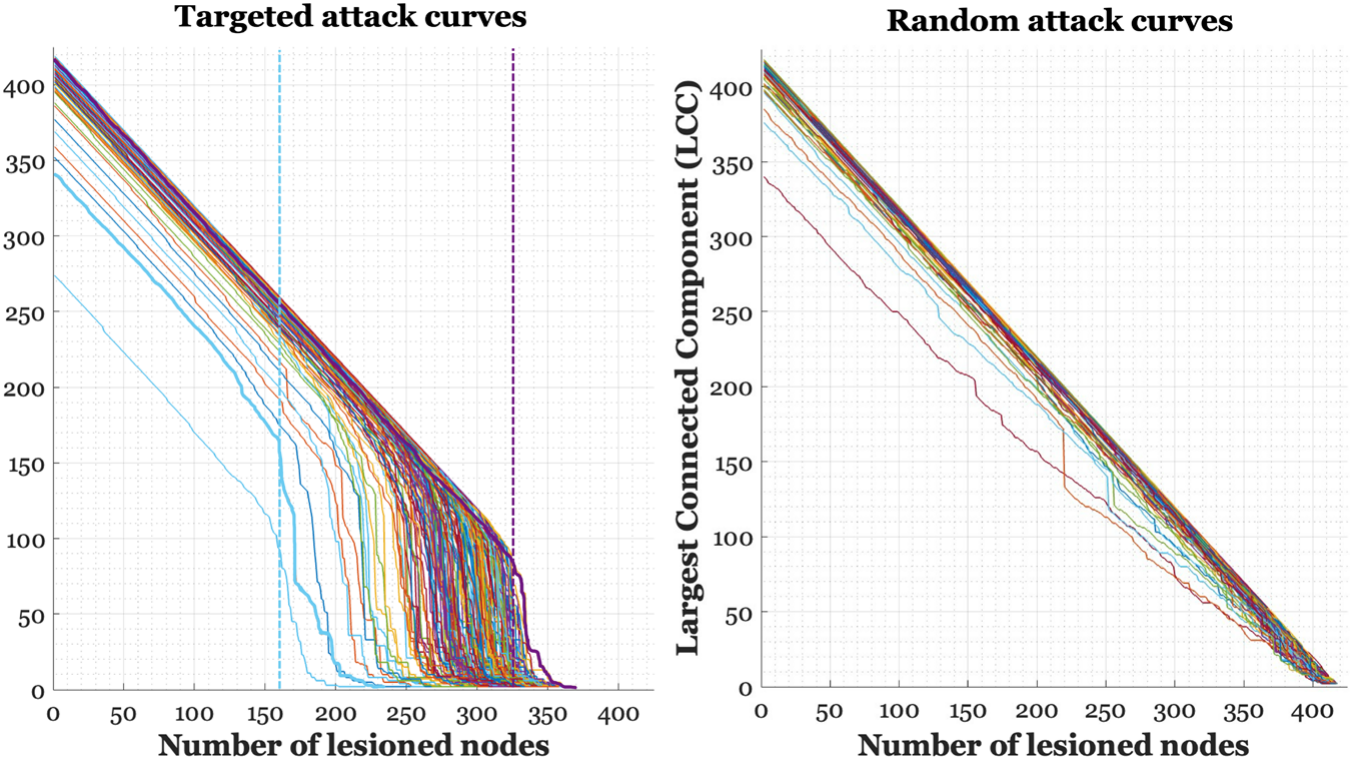
Line plots of each participant’s largest connected component (LCC) curve at each time point 2 (T2), for both targeted attack (left) and random attack (right). Vertical dashed lines in targeted attack plot indicate the largest slope of decay for two example participants, where the early slope drop (blue) suggests a less resilient individual than one with late slope drop (purple). Critically, we can see from the plot that the random attack curves show no discernible drop in the LCC, reflecting the absence of targeted disruption to highly central nodes and indicating a gradual, nonspecific breakdown of network integrity.

The main results are reported for the primary connectivity-density threshold of 10%. In anticipation of our findings, results for models using the 5% and 15% connectivity-density thresholds are reported as Supplementary Table ST1 for the FLUID domain. For a visualization of the LCC lesioning curves at follow-up, for both targeted and random attack, see Figure 2. Critically, we focused on the interaction between the $LCC_{{\text{drop}}^{\text{T}2}}$ and ΔCT in the behavioral models. The $LCC_{{\text{drop}}^{\text{T}2}}\;$ was first standardized to reduce multicollinearity between the two predictors and for comparability across models. Additionally, some evidence suggests that healthy structural and functional brain networks exhibit small-world topology (Stam, 2010), this property being integral to the study of network robustness (Albert et al., 2000; see Aerts et al., 2016), we investigated the relationship between small-world architecture of the original unlesioned graphs and both network resilience and behavioral performance. Bivariate correlations among the primary variables of interest are reported in Table 2.

**Table 2.** Correlation matrix displaying bivariate associations among the main study variables**.**

	ΔFLUID	Age	Sex	Edu	NART	ΔCT	$LCC_{{\text{drop}}^{\text{T}2}}$	$LCC_{\text{k}=0^{\text{T}2}}$	MConn_T2_	$SW_{\text{T}2}$	Scrub%_T2_	FLUID_T1_
ΔFLUID	—											
Age	-0.12	—										
Sex	0.17	-0.06	—									
Edu	0.03	0.09	-0.06	—								
NART	-0.06	0.01	-0.10	0.59	—							
ΔCT	0.18	-0.15	0.04	0.13	-0.12	—						
$LCC_{{\text{drop}}^{\text{T}2}}$	0.10	0.04	0.18	0.07	0.05	-0.08	—					
$LCC_{\text{k}=0^{\text{T}2}}$	0.12	0.05	0.15	0.07	0.01	0.01	0.83	—				
MConn_T2_	-0.04	-0.08	-0.16	-0.02	-0.04	0.06	-0.76	-0.68	—			
$SW_{\text{T}2}$	0.07	0.06	0.15	0.07	0.08	-0.05	0.87	0.62	-0.75	—		
Scrub%_T2_	0.04	0.20	-0.13	-0.05	-0.14	-0.16	-0.10	-0.13	0.09	-0.04	—	
FLUID_T1_	-0.38	-0.25	-0.17	0.28	0.58	-0.04	-0.10	-0.13	0.07	-0.06	-0.194	—

Correlations are presented with two decimal precision.

MConn = mean connectivity of matrix for 10% density threshold; SW = small-world property; CT = cortical thickness.

### 3.1 Behavioral models

**Fig. 3.**
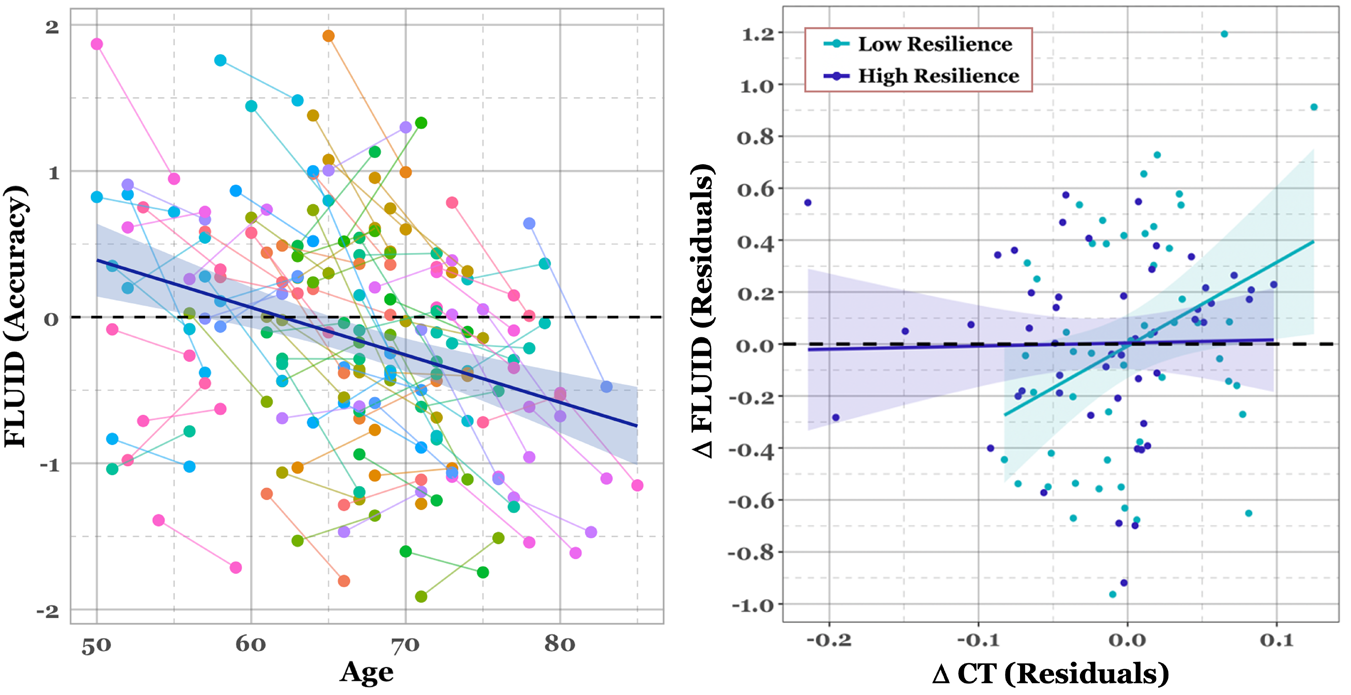
Scatterplots of longitudinal change in Fluid Reasoning (FLUID) performance. Left panel: Spaghetti plot of each individual’s fluid reasoning accuracy score at baseline and 5-year follow-up. Color assignments are random for better separability. The line and shaded ribbon represent the linear trend across all participants with the standard error of the fit, respectively. Right panel: Relationship between longitudinal change in Fluid Reasoning performance and CT, divided by network resilience (i.e., ${\text{LCC}}_{{\text{drop}}^{\text{T}2}}$ ). The change in fluid reasoning performance (Δ FLUID) represents the residuals after adjusting the model for all factors, excluding ΔCT and $LCC_{{\text{drop}}^{\text{T}2}}$ . ΔCT represents the residuals after adjusting change values (T2 − T1) for baseline CT. Resilience was determined by median split in standardized scores in $LCC_{{\text{drop}}^{\text{T}2}}$ .

We investigated the effect of demographic factors, brain integrity (ΔCT), and network resilience ($LCC_{{\text{drop}}^{\text{T}2}}$ ) on change in behavioral performance for each cognitive domain. We also controlled for scrubbing percentage at time 2 (Scrub%_T2_), initial LCC at time 2 ($LCC_{\text{k}=0^{\text{T}2}}$ ), and baseline behavioral performance. For a full list of predictors and parameters, see Table 3. For all cognitive domains, baseline performance significantly predicted the change in performance over time. For MEM, FLUID, and SPEED, this relationship was negative, such that higher baseline performance predicted less change over time. For VOCAB, this relationship was positive; however, there was also a significant negative effect of NART, such that higher NART predicted less change over time. Additionally, as can be observed from the model estimates, the standardized beta coefficients indicated potential multicollinearity, which was confirmed for the variables NART and baseline performance by variance inflation factor. Age was also significantly linked to longitudinal behavioral declines for MEM, FLUID, and SPEED, but not for the VOCAB domain. The most notable findings overall were for the FLUID domain, where both ΔCT and the interaction between ΔCT and the $LCC_{{\text{drop}}^{\text{T}2}}$ both significantly predicted change in fluid reasoning (ΔFLUID) over time. ΔCT was positively linked to behavioral change as expected, where higher brain integrity over time was related to better behavioral performance. Critically, there was a negative interaction between ΔCT and the $LCC_{{\text{drop}}^{\text{T}2}}$ , where individuals with higher network resilience were less sensitive to the effects of change in structural integrity on ΔFLUID (see Fig. 3 for plot of longitudinal Fluid Reasoning performance and interaction effect). Additionally, we conducted a Freedman–Lane permutation test, which directly evaluates the interaction under the null by fitting a reduced model without the interaction term, permuting the residuals from this model, and adding them back to the fitted values to generate permuted outcome vectors. These permuted outcomes were then refit with the full model (including the interaction term) to obtain the null distribution of the interaction t-statistic (1,000 permutations). The observed interaction was significant (t = –2.310, p = .019). As a robustness check, we also repeated the test by permuting the ΔFLUID dependent variable directly (a stricter null that removes all predictor–outcome associations), which produced a similar result (p = .008), supporting the robustness of the interaction finding to permutation strategy. This interaction between ΔCT and the $LCC_{{\text{drop}}^{\text{T}2}}$ held up when we considered (1) density thresholds of both 5% and 15%; (2) the shorter truncated time series at 10% density threshold; and (3) normalization of the LCC curves using LCC metrics derived from targeted attack performed on individual-level randomized matrices (see Supplementary Table ST1). To account for potential confounding by global connectivity levels, we repeated the moderation analysis with mean connectivity of the 10% density thresholded functional connectivity matrix included as an additional covariate. This adjustment ensures that the observed moderation effect is not attributable to overall differences in connectivity strength across individuals. The moderation effect remained significant after including this covariate (see Supplementary Table ST2).

**Table 3.** List of predictors for all linear regression behavioral models.

Outcome	Predictor	β	p	CI	$\eta_{\rho }^{2}$
ΔMEM	Age	**-.341**	<.001***	**[-.052 -.014]**	**.114**
	Edu	.162	.184	[-.024 .125]	.020
	Sex	.080	.408	[-.157 .382]	.008
	NART	-.053	.672	[-.028 .018]	.002
	MEM_T1_	**-.582**	<.001***	**[-.551 -.192]**	**.160**
	Scrub%_T2_	.098	.968	[-.020 .021]	.000
	$LCC_{{\text{drop}}^{\text{T}2}}$	-.101	.197	[-.407 .085]	.010
	$LCC_{\text{k}=0^{\text{T}2}}$	.133	.127	[-.005 .042]	.026
	ΔCT	.208	.837	[-2.358 2.904]	.001
	$LCC_{{\text{drop}}^{\text{T}2}}$ * ΔCT	-.218	.076	[-5.076 .261]	.035
ΔFLUID	Age	**-.250**	.011*	**[-.027 -.004]**	**.071**
	Edu	-.009	.939	[-.047 .044]	.000
	Sex	.098	.284	[-.075 .254]	.013
	NART	**.303**	.025*	**[.003 .036]**	**.056**
	FLUID_T1_	**-.582**	<.001***	**[-.465 -.202]**	**.223**
	Scrub%_T2_	.098	.337	[-.006 .018]	.011
	$LCC_{{\text{drop}}^{\text{T}2}}$	-.101	.553	[-.200 .108]	.000
	$LCC_{\text{k}=0^{\text{T}2}}$	.133	.429	[-.009 .021]	.007
	ΔCT	**.208**	.036*	**[.109 3.278]**	**.029**
	$LCC_{{\text{drop}}^{\text{T}2}}$ * ΔCT	**-.218**	.023*	**[-3.517 -.264]**	**.057**
ΔSPEED	Age	**-.343**	.002**	**[-.037 -.009]**	**.107**
	Edu	.171	.180	[-.017 .091]	.020
	Sex	.123	.224	[-.074 .314]	.017
	NART	.084	.525	[-.012 .022]	.005
	SPEED_T1_	**-.233**	.043*	**[-.308 -.005]**	**.045**
	Scrub%_T2_	.202	.054	[-.001 .029]	.041
	$LCC_{{\text{drop}}^{\text{T}2}}$	-.143	.451	[-.255 .114]	.008
	$LCC_{\text{k}=0^{\text{T}2}}$	.190	.302	[-.008 .027]	.012
	ΔCT	-.088	.419	[-2.659 1.116]	.006
	$LCC_{{\text{drop}}^{\text{T}2}}$ * ΔCT	.037	.727	[-1.614 2.305]	.001
ΔVOCAB	Age	-.069	.506	[-.012 .006]	.005
	Edu	.082	.539	[-.026 .050]	.004
	Sex	-.039	.695	[-.156 .105]	.002
	NART	**-1.205**	.002**	**[-.085 -.020]**	**.107**
	VOCAB_T1_	**.899**	.013*	**[.082 .661]**	**.069**
	Scrub%_T2_	.020	.846	[-.009 .011]	.000
	$LCC_{{\text{drop}}^{\text{T}2}}$	-.207	.269	[-.190 .054]	.021
	$LCC_{\text{k}=0^{\text{T}2}}$	.211	.252	[-.005 .018]	.015
	ΔCT	-.117	.291	[-1.970 .598]	.004
	$LCC_{{\text{drop}}^{\text{T}2}}$ * ΔCT	.186	.092	[-.192 2.517]	.032

Significant predictors are highlighted in bold. Change (Δ) in behavior of each domain (MEM = memory; FLUID = fluid reasoning; SPEED = processing speed; VOCAB= vocabulary) is the outcome variable. We also controlled for scrubbing (Scrub%_T2_), initial LCC ($LCC_{\text{k}=0^{\text{T}2}}$), and baseline behavioral performance in each model. Change in CT (ΔCT: T2 − T1) was residualized with respect to baseline. NOTE: The standardized beta coefficient exceeds one for the relationship between NART and ΔVOCAB due to high multicollinearity between VOCAB_T1_ and NART; model effects should be cautiously interpreted. Asterisks indicate statistical significance at threshold levels p < 0.05 (*), p < 0.01 (**), and p < 0.001(***).

β = Standardized coefficient beta; *p*= p-value (uncorrected); CI = 95% confidence interval; $\eta_{\rho }^{2}$ = partial eta-squared effect size.

To confirm that the $LCC_{{\text{drop}}^{\text{T}2}}$ served as a measure of CR beyond other network metrics, we also examined two additional network metrics—modularity-Q and system segregation (as defined by Chan et al., 2014). System segregation quantifies the relative strength of within- versus between-network connectivity. It is computed as the difference between the mean within-network and between-network connections, normalized by the mean within-network connectivity (Chan et al., 2014). Modularity Q measures how well a network can be divided into non-overlapping communities with dense internal and sparse external connections (Newman, 2006). Higher Q values indicate stronger community structure, with significance assessed against chance (Sporns & Betzel, 2016). When included as candidate CR measures, neither system segregation (p = .1332) nor modularity Q (p = .4045) significantly moderated the relationship between ΔCT and ΔFLUID (see Supplementary Table ST3). For completeness, we also examined whether baseline (T1) $LCC_{\text{drop}}$ or $\Delta LCC_{\text{drop}}$ moderated cognitive change. $LCC_{{\text{drop}}^{\text{T}1}}$ was not a significant moderator, whereas change in $\Delta LCC_{\text{drop}}$ from baseline to follow-up (controlling for baseline) significantly moderated the effect of ΔCT on ΔFLUID (see Supplementary Table ST4).

### 3.2 Relationship between small-world and network resilience

We investigated the relationship between static small-world property of each individual’s unlesioned matrix at follow-up (i.e., $SW_{\text{T}2}$ ) and $LCC_{{\text{drop}}^{\text{T}2}}$ . We found that both the initial LCC on the unlesioned matrix ($LCC_{\text{k}=0^{\text{T}2}}$ ) and $SW_{\text{T}2}$ both significantly positively predicted the $LCC_{{\text{drop}}^{\text{T}2}}$ ; that is, higher initial LCC and small-world property of the network was linked to higher resilience (see Table 4). When we added $SW_{\text{T}2}$ to the FLUID model to see if it accounted for any variance in ΔFLUID, the interaction between ΔCT and the $LCC_{{\text{drop}}^{\text{T}2}}$ remained significant (β = -.217, 95% CI [-.126 -.009], p = .024, $\eta_{\rho }^{2}$ **=** 0.057).

Accounting for baseline differences and covariates, we found a significant negative interaction between network resilience and ΔCT on ΔFluid Reasoning performance. Our finding supports evidence for targeted attack as a measure of CR, where higher brain network resilience may have permitted individuals with reduced structural brain integrity to better cope with structural loss by enhance preservation of cognitive function.

**Table 4.** List of predictors for the model with ${\text{LCC}}_{{\text{drop}}^{\text{T}2}}$ as outcome.

Outcome	Predictor	β	p	CI	$\eta_{\rho }^{2}$
${\text{LCC}}_{{\text{drop}}^{\text{T}2}}$	Age	-.027	.474	[-.0396 .184]	.006
	Edu	-.003	.939	[-1.115 1.032]	.0001
	Sex	.023	.492	[-2.457 5.07]	.005
	NART	.005	.927	[-.308 .337]	.0001
	$LCC_{{\text{drop}}^{\text{T}1}}$	.033	.371	[-.045 .119]	.009
	Scrub%_T2_	-.007	.847	[-.309 .254]	.0004
	$LCC_{\text{k}=0^{\text{T}2}}$	**.461**	<.001***	**[1.022 1.511]**	**.541**
	CT_T2_	-.005	.892	[-21.904 19.102]	.0002
	$SW_{\text{T}2}$	**.575**	<.001***	**[25.915 34.77]**	**.673**

Significant predictors are highlighted in bold. We also controlled for scrubbing (Scrub%_T2_) and initial LCC ($LCC_{\text{k}=0^{\text{T}2}}$). Asterisks indicate statistical significance at threshold levels p < 0.001(***).

β = Standardized coefficient beta; p = p-value (uncorrected); CI = 95% confidence interval; $\eta_{\rho }^{2}$ = partial eta-squared effect size.

### 3.3 Critical node maps

**Fig. 4.**
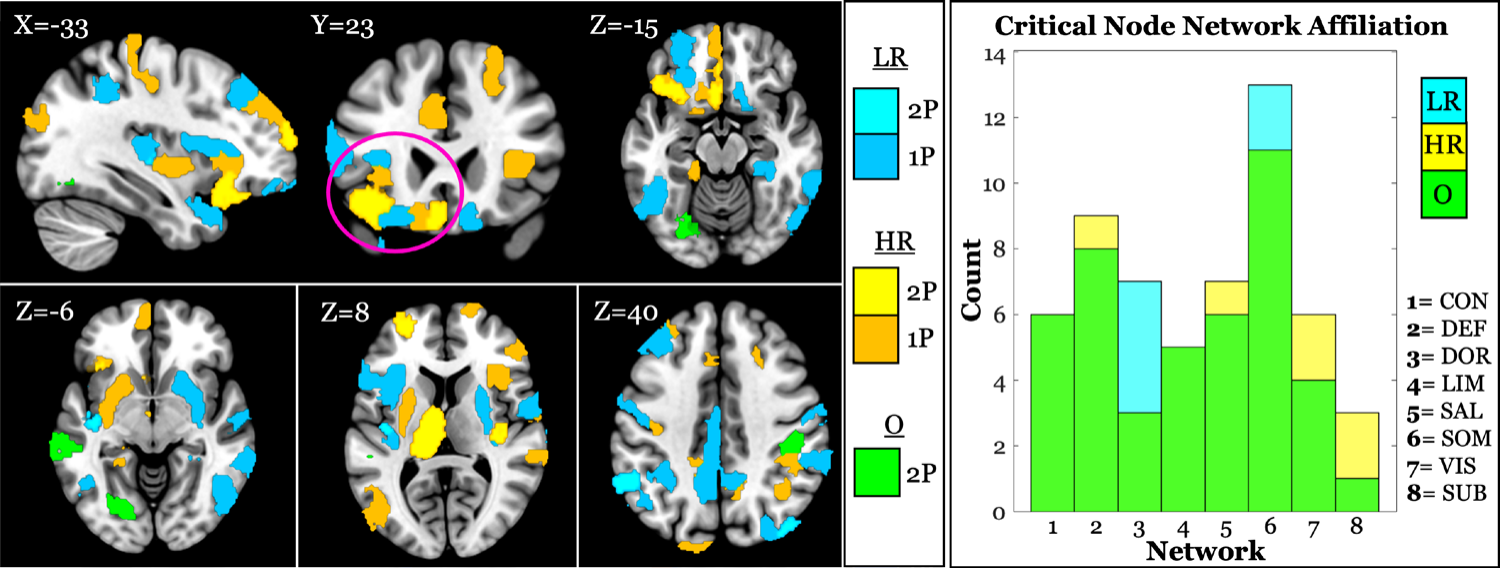
Spatial distribution and network affiliation of the critical node of LCC drop across individuals, grouped by network resilience. Left: For each participant, the brain region whose removal corresponded to the largest decline in the largest connected component (LCC) was identified and mapped in MNI space. Frequency maps display the location of these critical nodes for low resilience (LR) and high resilience (HR) groups, based on a median split of the critical drop iteration, along with any regional overlap (O) between groups. Within each group, regional overlap was limited to either one (1P) or two (2P) participants per region. While some spatial patterns emerged (e.g., greater parietal involvement in low resilience, more frontal regions in high resilience), overall overlap was low, highlighting substantial inter-individual variability in network vulnerability. However, a cluster of critical nodes emerged in the left frontal cortex (pink circle). Right: Histogram of network affiliation for the critical node of LCC drop. Network abbreviations are: CON = control; DEF = default mode; DOR = dorsal attention; LIM = limbic; SAL = salience; SOM = somatosensory; VIS = visual; SUB = subcortical. NOTE: Subcortical nodes are anatomically defined and not by functional network organization.

To characterize the spatial distribution of network vulnerability, we identified the *critical node of drop*—the node whose targeted removal corresponded to the sharpest decline in the LCC—for each participant. Participants were divided into low and high resilience groups based on a median split of their critical drop iteration. Frequency maps of these critical nodes were generated in MNI space and visualized to examine group-level patterns (see Fig. 4). The spatial distribution of critical nodes indicated that low resilience participants most commonly showed critical nodes in parietal regions, including the bilateral angular gyrus, inferior parietal cortex, supramarginal gyrus, postcentral gyrus, and midcingulate cortex. In contrast, high resilience participants exhibited a greater concentration of critical nodes in frontal regions, such as the superior and middle frontal gyrus and orbitofrontal cortex, bilaterally. Notably, a left-lateralized cluster—including the inferior and superior orbitofrontal cortex, insula, rectus, and adjacent middle frontal cortex—appeared as a shared vulnerability zone across both groups, though more frequently observed in the high resilience group. However, these observations are based on visual inspection of group-level maps and do not indicate a clear, consistent anatomical pattern across individuals. This variability highlights the importance of considering individual-level differences in network topology when characterizing resilience. High variability in network affiliation was also observed; however, the most notable difference emerged in the dorsal attention network, where a greater number of low-resilience individuals (7) exhibited a critical node of LCC drop compared to high-resilience individuals (3). While this may suggest a greater reliance on the dorsal attention network in low-resilience individuals, the small absolute difference warrants cautious interpretation.

## 4 Discussion

In the present paper, our main objective was to investigate whether greater network resilience, as assessed via targeted attack of resting-state functional connectivity networks, acted as a mechanism of CR among older age adults. We utilized the LCC of the network as our topological feature of interest, identifying the critical point of LCC drop, which served as our measure of resilience; this measure has frequently been employed in the literature when quantifying the robustness of a network under targeted, sustained attack (Albert et al., 2000, Albert & Barabási, 2002; Joyce et al., 2013; Menardi et al., 2021). Critically, we observed a moderation effect of this critical point of LCC drop on the relationship between structural brain changes and cognitive performance changes notably for the fluid reasoning domain. To our knowledge, this is the first study to identify a metric of targeted attack that functioned as a mechanism of CR, in line with the recent framework established for the study of resilience and reserve (Stern et al., 2023).

Prior work in simulated network attack has typically aimed to compare network robustness under different attack strategies to infer to what extent a biological network displays specific architectures such as scale-free, small-world, and random networks (Achard et al., 2006; Albert et al., 2000, 2002). Targeted attacks are often compared to random attacks to evaluate the robustness and vulnerability of the network. A random attack involves the arbitrary removal of nodes or edges, representing random failures or disruptions. In contrast, targeted attack specifically removes the most critical nodes, such as those with the highest degree, strength, or centrality, simulating a strategic assault on the network’s most influential components. Comparing these different attacks offers insight into underlying network structure and resilience patterns; whereas small-world topology, for instance, is typically robust to random attacks, given its distributed and highly clustered configuration, it is more vulnerable to targeted attack of its hubs. In the present work, our aim was not to systematically compare targeted versus random attack as a means of confirming or refuting canonical network structure, as has been done in foundational studies using synthetic or idealized networks and topological metrics (e.g., Albert et al., 2000; Watts & Strogatz, 1998), but rather to directly examine individual differences in network resilience to targeted attack and to elucidate their relationship to structural and cognitive changes. Nonetheless, to visually contextualize our findings and rule out the possibility that the observed effects reflect generic network vulnerabilities, we performed a random attack procedure. As expected, this resulted in a gradual, linear decline in LCC size with no sharp inflection point (see Fig. 2), supporting the interpretation that the abrupt breakdown observed in the targeted attack reflects selective vulnerability to the removal of high-strength nodes—a form of weighted centrality—rather than general network fragility. This analysis was conducted for visualization and interpretive purposes only; random attack metrics were not included in statistical modeling, as our focus was specifically on the network’s resilience to targeted, biologically meaningful disruption. Given the well-established evidence for the small-world topology of brain networks (see Bassett & Bullmore, 2017; Watts & Strogatz, 1998), we further investigated the relationship between the LCC drop and small-world properties of the unlesioned connectivity matrix. Regression analysis revealed that both the initial LCC and the small-worldness of the unlesioned matrix independently and positively predicted the LCC drop. Prior work has, indeed, shown that small-world topology is integral to the study of network robustness (Aerts et al., 2016; Albert et al., 2000); this architecture enhances synchronization and information flow, while supporting diverse neural computations by integrating local processing across distributed networks (Bassett & Bullmore, 2017).

The fact that network resilience moderated the relationship between structural brain changes and cognitive changes, specifically in the Fluid Reasoning domain, is particularly noteworthy. Prior work in the lab examined changes in the number of graph components across various connectivity filtration thresholds as an indicator of network integrity. The findings showed that a longer transition from isolated nodes to a single connected component was positively associated with better longitudinal performance in fluid reasoning over time (Argiris et al., 2023). Interestingly, this finding was observed in task-based functional connectivity; here, we find a relationship between higher network resilience during resting-state connectivity and out-of-scanner neuropsychological task performance. Some work has supported the notion that task-based functional connectivity better predicts both cognitive task activations (Cole et al., 2021) and as well as cognitive performance when studied in the context of a global neural flexibility (Varangis et al., 2022). It could be that the relationship between resting-state functional connectivity and cognition may depend on both the network property and/or the cognitive domain being measured. Notably, higher fluid intelligence has been linked to more efficient brain connectivity updates in network reconfiguration, indicating smaller changes in functional network architecture between task and rest (Schultz & Cole, 2016). It is important to highlight the distributed nature of networks underlying fluid reasoning processing (Colom et al., 2010). Given the robust association between fluid reasoning and whole-brain network metrics, it may be particularly insightful to focus on fluid reasoning when studying both healthy and potentially pathological aging.

While group-level visualizations of the critical nodes of LCC drop suggested that individuals with lower network resilience tended to show early topological disintegration in parietal regions (e.g., angular gyrus, supramarginal gyrus, midcingulate), and those with higher resilience more often in frontal regions (e.g., orbitofrontal cortex, middle frontal gyrus), these findings should be interpreted with caution. Across participants, the maximum overlap in any given region was limited to only two individuals, underscoring the high degree of variability in the topography of critical nodes. This heterogeneity aligns with emerging perspectives emphasizing the importance of individualized brain network profiles in predicting cognitive outcomes (Gordon et al., 2017; Mueller et al., 2013) and cautions against over-interpreting group-level spatial patterns. While the observed regions are known to support integrative functions such as attention, control, and valuation (Andrews-Hanna et al., 2014; Rolls, 2019), the dispersed and individually specific nature of the findings highlights the value of approaches that model network resilience at the individual level rather than relying solely on spatial overlap or common regional vulnerabilities. Additionally, a slightly higher incidence of critical node in the dorsal attention network among low-resilience individuals may reflect increased functional reliance on this network at rest, aligning with prior evidence linking attentional network connectivity to cognitive reserve in aging (Franzmeier et al., 2017); however, given the modest group difference, this observation should be interpreted with caution, and we did not assess the specific network connections of the critical node at the point of network disintegration, limiting conclusions about whether its role reflects within-network vulnerability or broader cross-network integration.

One important point of consideration is the fact that our network resilience measure was calculated on weighted graphs that were thresholded to retain the strongest connections. Despite this being a common practice in the literature (Bullmore & Sporns, 2009; Van Wijk et al., 2010), more recent work has argued for the importance of considering weak connections in neural systems (Bassett & Bullmore, 2017). Importantly, some work has shown that individual differences in the strength of weak functional connections from the prefrontal cortex to the frontoparietal network predict differences in fluid intelligence (Cole et al., 2012). Our decision to threshold the weighted matrices was due to the limited variability in our network measure of consideration (i.e., LCC) when no threshold was applied. To better capture individual differences in network resilience while still maintaining robust analytical validity, we evaluated the consistency of our findings across different threshold levels; our findings upheld across all thresholds. Additionally, we repeated the analysis with the truncated time series, again replicating our original findings; this is critical, despite work showing that greater reliability is found at longer scan lengths (Birn et al., 2013). However, it is important to note that our primary moderation analyses relied on LCC drop at follow-up. Although this limits the ability to make prospective claims, it remains consistent with how CR is typically operationalized: traditional proxies such as education or IQ are generally assessed once and treated as stable indicators of reserve rather than modeled longitudinally. Using the LCC drop at follow-up provided a later-life, brain-based ‘snapshot’ of resilience and the longer resting-state acquisition at follow-up afforded greater reliability of the functional connectivity measure. However, to further address this issue, we conducted supplementary analyses testing baseline and change in LCC drop (see ST4). While baseline values did not significantly moderate cognitive change, the change in LCC drop from baseline to follow-up did, supporting the notion that dynamic, brain-based indices of resilience may capture aspects of reserve beyond static measurement. Together, these findings suggest that while predictive utility cannot be fully established here, our approach offers a valid and informative proxy of CR.

In the present work, we also only considered the point of drop in the LCC, via successive nodal removal, as our measure of network resilience. Other work has focused not only on other nuanced elements of the lesioning curve, but also on targeted edge removal as a potentially more sensitive measure of resilience (Menardi et al., 2021). Other metrics across different topological scales, in addition to quantifying resilience in specific networks, may also serve as viable indicators of network disruption, particularly when comparing aging to AD pathology (Fathian et al., 2022; Watanabe et al., 2021). Additionally, while real-world lesion studies have shown that the effects of damage to brain networks vary depending on the specific regions (Warren et al., 2014) and types of connections involved (e.g., “hub” versus “connector”; Gratton et al., 2012), our in-silico lesioning approach is designed to evaluate network vulnerability from a topological perspective. We acknowledge that functional outcomes in real patients are shaped by multiple interacting factors, including functional redundancy, regional specialization, and individual variability. However, connection strength remains a fundamental organizing principle for understanding network robustness. Prior work in biological, communication, and neural networks has shown that removing high-strength or high-degree nodes can critically disrupt network integrity (Albert et al., 2000; Bullmore & Sporns, 2009; Menardi et al., 2021; Watts & Strogatz, 1998). While our approach does not simulate focal lesions per se, it provides a principled framework to probe how the loss of strongly connected regions may affect global network organization, while still accounting for individual variability in network architecture. Thus, this method complements lesion studies by providing insight into general principles of network vulnerability and robustness that may underlie observed clinical outcomes. Finally, our data were collected with eyes closed. Although some studies have reported differences between eyes-closed and eyes-open acquisitions, Patriat et al. (2013) observed reduced reliability only in the visual network, with no significant differences in default-mode, attention, or motor networks. Other work has shown comparable or even stronger connectivity under eyes-closed conditions (e.g., Han et al., 2023). As there is no unified standard in the field, and all participants were scanned under the same condition, any influence is likely systematic across the sample rather than differential across groups.

As a next step, we plan to test the generalizability of the present findings in additional cohorts, including samples of older individuals progressing along the AD spectrum. This will include evaluating the predictive utility of our measures in identifying conversion from cognitively healthy status to mild cognitive impairment (MCI), as well as from MCI to AD. Additionally, transcranial magnetic stimulation (TMS) may be used to probe dynamic network reconfiguration in response to targeted disruption. Although TMS does not offer the spatial precision of in silico lesioning, which can simulate perturbations at high resolution using detailed atlases, it provides a complementary approach for examining how brain networks respond to disruption of theory-driven targets—such as hubs—and assessing phenomena like network rebound.

In summary, our study identifies a novel functional network resilience metric—defined by the critical point of LCC drop during targeted attack—as a potential mechanism of cognitive reserve in aging. We found that this metric moderated the relationship between structural brain changes and changes in fluid reasoning ability, suggesting that individuals with more robust functional network organization are better able to maintain cognitive performance despite age-related neural decline. By linking individualized network vulnerability profiles to cognitive outcomes, our findings contribute to a growing literature emphasizing the importance of connectome-based markers of resilience. These findings highlight the value of topologically grounded connectome-based markers for understanding individual differences in cognitive aging and support future efforts to identify at-risk individuals and assess resilience in clinical populations, including AD.

## Ethics

The Columbia University Institutional Review Board approved all study procedures.

## Supplementary Material

Supplementary Material

## Data Availability

The data that support the findings of this study are available from the corresponding author upon reasonable request. Custom-written code detailing analysis can also be made available upon reasonable request.
