# Supplementary material for: Brain resilience to targeted attack of resting BOLD networks as a measure of cognitive reserve
Source: Imaging Neurosci (Camb). 2026 Jan 13;4:IMAG.a.1065. doi: 10.1162/IMAG.a.1065 (PMC12801055; doi:10.1162/IMAG.a.1065)
Supplement: Supplementary Material [file IMAG.a.1065_supp.pdf]

Supplemental Material

	Predictor	β	***p***	***CI***	η_{ρ}^{2}
**5% Density Threshold**	Age	-.242	.015*	[-.027 -.003]	.066
	Edu	-.018	.878	[-.049 .042]	.0003
	Sex	.089	.335	[-.085 .246]	.011
	NART	.328	.016*	[.004 .038]	.064
	FLUID$_{T1}$	-.612	<.001***	[-.483 -.217]	.237
	Scrub%$_{T2}$	.085	.382	[-.007 .018]	.009
	$LCC_{drop^{T2}}$	-.180	.316	[-.245 .080]	.007
	$LCC_{k=0^{T2}}$	.176	.327	[-.003 .009]	.011
	ΔCT	.186	.055	[-.031 3.053]	.031
	$LCC_{drop^{T2}}$ * ΔCT	-.187	.046*	[-3.329 -.035]	.045
**15% Density Threshold**	Age	-.237	.015*	[-.027 -.003]	.065
	Edu	-.027	.811	[-.051 .040]	.001
	Sex	.115	.214	[-.062 .271]	.017
	NART	.329	.015*	[.004 .038]	.065
	FLUID$_{T1}$	-.580	<.001***	[-.464 -.200]	.220
	Scrub%$_{T2}$	.105	.275	[-.006 .019]	.014
	$LCC_{drop^{T2}}$	-.120	.363	[-.174 .064]	.001
	$LCC_{k=0^{T2}}$	.183	.162	[-.006. .036]	.022
	ΔCT	.215	.030*	[.179 3.320]	.032
	$LCC_{drop^{T2}}$ * ΔCT	-.221	.024*	[-3.726 -.270]	.057
**Truncated Time Series (10% Density Threshold)**	Age	-.244	.012*	[-.027 -.003]	.070
	Edu	-.013	.910	[-.048 .043]	.0001
	Sex	.094	.301	[-.077 .247]	.012
	NART	.307	.021*	[.003 .036]	.059
	FLUID$_{T1}$	-.599	<.001***	[-.474 -.212]	.235
	Scrub%$_{T2}$	.068	.466	[-.008 .017]	.006
	$LCC_{drop^{T2}}$	-.041	.808	[-.169 .132]	.002
	$LCC_{k=0^{T2}}$	.020	.905	[-.017 .020]	.0002
	ΔCT	.175	.072	[-.132 2.986]	.025
	$LCC_{drop^{T2}}$ * ΔCT	-.257	.006**	[-3.687 -.633]	.082
**Normalized LCC (10% Density Threshold)**	Age	-.236	.017*	[-.027 -.003]	0.017
	Edu	.015	.899	[-.043 .049]	0.899
	Sex	.101	.274	[-.074 .256]	0.274
	NART	.298	.027*	[.002 .035]	0.027
	FLUID$_{T1}$	-.590	.000***	[-.47 -.206]	0.000
	Scrub%$_{T2}$	.065	.500	[-.008 .017]	0.500
	norm$LCC_{drop^{T2}}$	.006	.951	[-.081 .087]	0.767
	norm$LCC_{k=0^{T2}}$	.070	.451	[-0.052 .116]	0.451
	ΔCT	.215	.032*	[0.156 3.337]	0.102

	norm$LCC_{\mathrm{drop}^{\mathrm{T2}}}$ * ΔCT	-.198	.037*	[-3.292 -.108]	0.037

ST1. List of predictors for linear regression models with ΔFLUID as the outcome variable. We report the results for (1) 5% density thresholding of the FC matrices; (2) 15% density thresholding of the FC matrices; (3) when the 5-minute truncated time series with 10% density thresholding; and (4) with $LCC_{\mathrm{drop}^{\mathrm{T2}}}$ normalized (norm) by the LCC curves derived from randomized matrices. Again, we controlled for scrubbing (Scrub%$_{\mathrm{T2}}$), initial LCC ($LCC_{\mathrm{k=0}^{\mathrm{T2}}}$), and baseline behavioral performance in each model. Change in CT (ΔCT: T2 – T1) was residualized with respect to baseline. Asterisks indicate statistical significance at threshold levels p<0.05 (*), p<0.01 (**), and p<0.001(***).
β= Standardized coefficient beta; *p*= p-value (uncorrected); *CI*= 95% confidence interval; $\eta_\rho{}^2$ = partial eta-squared effect size.

	Predictor	***β***	***p***	***CI***	$\boldsymbol{\eta_\rho{}^2}$
**10% Density Threshold (mean connectivity in model)**	Age	-.260	.009**	[-.028 -.004]	.076
	Edu	-.010	.927	[-.047 .043]	.000
	Sex	.067	.465	[-.104 .225]	.006
	NART	.335	.014*	[.004 .038]	.067
	FLUID$_{\mathrm{T2}}$	-.605	.000***	[-.478 -.215]	.241
	Scrub%$_{\mathrm{T2}}$	.143	.135	[-.004 .026]	.026
	$LCC_{\mathrm{drop}^{\mathrm{T2}}}$	-.056	.761	[-.195 .143]	.000
	$LCC_{\mathrm{k=0}^{\mathrm{T2}}}$	.169	.319	[-.007 .022]	.011
	ΔCT	.182	.061	[-.072 3.041]	.022
	Mean Connectivity$_{\mathrm{T2}}$	.134	.337	[-.983 2.844]	.011
	$LCC_{\mathrm{drop}^{\mathrm{T2}}}$ * ΔCT	-.234	.016	[-3.67 -0.386]	.065

ST2. List of predictors for linear regression models with ΔFLUID as the outcome variable, $LCC_{drop^{T2}}$ as predictor metric, and mean connectivity at 10% threshold included in the model. The moderation effect remained significant even after adjusting for mean connectivity. We controlled for scrubbing (Scrub%$_{\mathrm{T2}}$), initial LCC ($LCC_{\mathrm{k=0}^{\mathrm{T2}}}$), and baseline behavioral performance in each model. ΔCT was residualized with respect to baseline. Asterisks indicate statistical significance at threshold levels p<0.05 (*), p<0.01 (**), and p<0.001(***).
β= Standardized coefficient beta; *p*= p-value (uncorrected); *CI*= 95% confidence interval; $\eta_\rho{}^2$ = partial eta-squared effect size.

Variable	Wig's System segregation (LCC_{drop})	Modularity Q (LCC_{drop})
Age	-0.0082	-0.0157
Edu	0.6513	0.8024
Sex	0.4230	0.3625
NART	0.0298	0.0277
ΔCT	-0.2738	-0.7421
Network Metric $_{T2}$	0.4940	0.7974
Scrub% $_{T2}$	0.4825	0.7058
FLUID $_{T1}$	-0.0000	-0.0000
**Network Metric $_{T2}$ * ΔCT**	0.1332	0.4045

ST3. List of predictors for linear regression models with system's segregation and modularity-Q as network metrics and ΔFLUID as outcome measure. *Metrics were calculated at the critical point of $LCC_{\mathrm{drop}^{T2}}$. Values represent signed p-values, uncorrected. As can be observed from the table, neither system segregation nor modularity-Q displayed a significant interaction with ΔCT in explaining ΔFLUID.*

	Predictor	β	**p**	***CI***	η_ρ^2
**Baseline (T1)** LCC_{drop}	Age	-.286	.006**	[-.030 -.005]	.084
	Edu	.021	.854	[-.042 .051]	.000
	Sex	.069	.467	[-.107 .232]	.006
	NART	.333	.017*	[.004 .038]	.063
	FLUID $_{T1}$	-.616	.000***	[-.489 -.216]	.230
	Scrub% $_{T1}$	.131	.177	[-.005 .025]	.021
	$LCC_{\mathrm{drop}^{T1}}$	.163	.343	[-.080 .228]	.011
	$LCC_{\mathrm{k=0}^{T1}}$	-.147	.394	[-.033 .013]	.008
	ΔCT	.138	.150	[-.414 2.665]	.022
	$LCC_{\mathrm{drop}^{T1}}$ * ΔCT	.043	.646	[-1.299 2.085]	.002
**Change (Δ; T2–T1)** LCC_{drop}	Age	-.235	.015*	[-.026 -.003]	.065
	Edu	-.001	.994	[-.045 .045]	.001
	Sex	.108	.237	[-.066 .262]	.017
	NART	.279	.036*	[.001 .034]	.065
	FLUID $_{T1}$	-.560	.000***	[-.454 -.186]	.220
	ΔScrub%	.087	.358	[-.007 .019]	.014
	$\Delta LCC_{\mathrm{drop}}$	-.113	.420	[-.007 .003]	.001
	$\Delta LCC_{\mathrm{k=0}}$	.128	.349	[-.008 .021]	.022
	ΔCT	.238	.019*	[.326 3.545]	.032
	$\Delta LCC_{\mathrm{drop}}$ * ΔCT	-.266	.006**	[-.153 -.026]	.057

ST4. List of predictors for linear regression models with ΔFLUID as the outcome variable and baseline LCC_{drop} and change in LCC_{drop} are predictors. Treatment of the functional connectivity (FC) data is indicated in the green column. We report the results for (1) baseline (time point 1; T1) LCC_{drop}; (2) change (Δ) LCC_{drop} from baseline to follow-up (T2 – T1). Model was controlled for scrubbing (Scrub%), initial LCC ($LCC_{\mathrm{k=0}}$), and baseline behavioral performance (FLUID) in each model. Change in CT (ΔCT: T2 – T1) was residualized with respect to baseline. Asterisks indicate statistical significance at threshold levels p<0.05 (*), p<0.01 (**), and p<0.001(***).
β= Standardized coefficient beta; *p*= p-value (uncorrected); *CI*= 95% confidence interval; ${\eta_\rho}^2$ = partial eta-squared effect size.
